# Circulating MicroRNA-505 May Serve as a Prognostic Biomarker for Hypertension-Associated Endothelial Dysfunction and Inflammation

**DOI:** 10.3389/fcvm.2022.834121

**Published:** 2022-04-29

**Authors:** Qinbo Yang, Peiwei Wang, Yiqing Cai, Yimeng Cui, Jingang Cui, Xiaoye Du, Yu Chen, Teng Zhang

**Affiliations:** ^1^Yueyang Hospital of Integrated Traditional Chinese and Western Medicine, Shanghai University of Traditional Chinese Medicine, Shanghai, China; ^2^Clinical Research Institute of Integrative Medicine, Shanghai Academy of Traditional Chinese Medicine, Shanghai, China; ^3^Laboratory of Clinical and Molecular Pharmacology, Yueyang Hospital of Integrated Traditional Chinese and Western Medicine, Shanghai University of Traditional Chinese Medicine, Shanghai, China

**Keywords:** hypertension, endothelial dysfunction, inflammation, microRNA-505, prognostic biomarker

## Abstract

Our previous study has reported that the plasma microRNA-505 (miR-505) is elevated in hypertensive patients. However, the pathophysiological significance of miR-505 in hypertension remains to be elucidated. Hypertension is not only a vascular disorder, but also an inflammatory condition. The current study therefore aims to further investigate the pathophysiological implications of miR-505 in hypertension-associated vascular and inflammatory changes. *In vivo* experiments reveal that the plasma level of miR-505 is elevated in spontaneously hypertensive rats and angiotensin II-infused mice. In addition, miR-505 agomir treatment results in elevated blood pressure, endothelial dysfunction, increased vascular expression of inflammatory genes and renal inflammatory injuries as well as pre-activation of PBMCs in mice. *In vitro* experiments further demonstrate that miR-505 agomir increases the expression of *IL1B* and *TNFA*, whereas miR-505 antagomir attenuates TNF-α-induced upregulation of *IL1B* and *TNFA* in endothelial cells, HUVECs. In addition, miR-505 modulates the levels of endothelial activation markers VCAM1 and E-selectin in HUVECs as well as the adhesion of THP-1 monocytes to HUVECs. Lastly, the plasma level of miR-505 is positively correlated with systolic blood pressure and the level of C-reactive protein in human subjects. Our work links for the first time miR-505 to endothelial dysfunction and inflammation under hypertensive conditions, supporting the translational value of miR-505 in prognosticating hypertension-associated endothelial impairment and inflammatory injuries in target organs such as the vessels and kidneys.

## Introduction

Long-term hypertension causes target organ damages in the heart, brain and kidney, etc. Mechanism-guided early detection and risk prediction of hypertensive target organ damages may help improve the clinical control of hypertension-associated target organ complications. Hypertension is closely associated with endothelial dysfunction (1). Chronic low-grade inflammation not only perpetuates the hypertensive state, but also promotes and aggravates hypertensive target organ damages (2, 3). Therefore, hypertension is not only a vascular disorder, but also an inflammatory condition. Our previous study has revealed that the plasma level of microRNA-505 (miR-505) is elevated in hypertensive patients. Our study has also demonstrated that miR-505 impairs the angiogenic potential of endothelial cells, suggesting the functional implications of miR-505 in endothelial cell biology (4). Meanwhile, it has been shown that dysregulated miR-505 is associated with the inflammatory phenotype of monocyte-derived macrophages in familial hypercholesterolemia (5). However, the pathophysiological significance of miR-505 in hypertension-associated vascular and inflammatory changes remains to be elucidated.

MicroRNAs (miRNAs) are short non-coding RNAs that post-transcriptionally regulate the expression of multiple functionally-related genes, playing important roles in a broad range of developmental, biological and pathophysiological processes (6). Circulating miRNAs are not only capable of exerting regulatory functions, but also highly accessible and remarkably stable. Therefore, circulating miRNAs have emerged as potential biomarkers for various pathological processes including cardiovascular diseases (7–9). Understanding the pathophysiological implications of circulating miR-505, specifically in hypertension-associated vascular and inflammatory alterations, may help develop accessible tools for the mechanism-based early detection and precise prognostication of hypertensive target organ damages.

## Materials and Methods

### Reagents

Phenylephrine (PE) was ordered from Tokyo Chemical Industry (Japan). Acetylcholine (Ach) and lipopolysaccharide (LPS) were purchased from Sigma-Aldrich (United States). Recombinant human tumor necrosis factor-α (TNF-α) protein was obtained from R&D Systems (United States). MiR-505 agomir (agomir-505), miR-505 antagomir (antagomir-505), negative control agomir (agomir-NC) and negative control antagomir (antagomir-NC) were synthesized by GenePharma (China).

### Animals and Treatment

Seventy-three 6-week-old C57BL/6J male mice (Shanghai Laboratory Animal Research Center, China) were used for the indicated experiments. For agomir-505 treatment, the mice received tail vein injection of agomiR-505 at the dose of 2.5 mg/kg body weight (bw) (low-dose) or 10 mg/kg bw (high-dose). The tail vein injection was performed every other day during the 2-week treatment. For controls, C57/BL6 mice were treated with agomir-NC (10 mg/kg bw) in the same manner. Whole blood, aorta or kidney specimens were collected for the indicated analyses. The blood pressure measurement and vascular reactivity assessment were performed 1 day, 1 week or 2 weeks after the 2-week injection. For gene expression analyses, the mice were euthanized 1 week after the agomir treatment. Immunohistochemical examination was performed 2 weeks after the termination of the agomir administration. Eight-week-old male C57BL/6J male mice (Shanghai Laboratory Animal Research Center, China) were subject to angiotensin II (Ang II) infusion (1 μg/kg/min) using osmotic mini-pumps (*n* = 5) (Alzet Model 1002, United States) for 2 weeks. Age- and sex-matched C57/BL6 mice implanted with the osmotic mini-pumps containing saline solution served as the sham controls (*n* = 5). Eight-week-old male spontaneously hypertensive rats (SHRs) (*n* = 6) and their age- and sex-matched strain control Wistar-Kyoto rats (WKY) (*n* = 6) (Beijing Vital River Laboratory Animal Technology Co., China) were also used for the indicated analyses. Along with their respective controls, Ang II-infused mice and SHRs were subject to the measurement of blood pressure. Whole blood from Ang II-infused mice and SHRs as well as their controls was also collected, followed by centrifugation at 1,000 rpm for 10 min to isolate the plasma. The plasma was analyzed to assess the level of circulating miR-505. The animal study was approved by the Institutional Animal Care and Use Committee at Yueyang Hospital, Shanghai University of Traditional Chinese Medicine. All the animal handling and procedures followed National Institutes of Health Guide for the Care and Use of Laboratory Animals.

### Blood Pressure Measurement

Blood pressure was measured by a non-invasive tail-cuff method using the BP-98E instrument (Softron Biotechnology, China). Briefly, conscious animals were restrained in a 40°C warming chamber and stabilized for 5–10 min. The cuff was then placed on the tail, followed by repeated inflation and deflation to condition the animals prior to acquiring the readings of systolic blood pressure (SBP). For each measurement, at least 10 readings within the 5–10 mm Hg range were taken.

### Vascular Reactivity Assay

Vascular reactivity was assessed using the DMT multiwire myograph system (620 M, Danish Myo Technology A/S, Denmark). Briefly, the aortic rings (3 mm in length) were equilibrated for 60–90 min and stimulated with 60 mM KCl for at least 3 times to verify the functional integrity of the vessel. After re-equilibration, the aortic rings were contracted with 1 μM PE to obtain a reproducible maximal contractile response. Vasodilation response to Ach was subsequently assessed by adding Ach at cumulative amounts at a 2-min interval with the final bath concentrations ranging from 10^–9^ M to 10^–5^ M. Vascular relaxation was calculated according to (N_PE_-N_Ach_)/(N_PE_-N_equilibration_).

### Flowcytometry Analysis

The kidneys were subject to collagenase digestion at 37°C for 30 min, followed by red blood cell lysis. Cells were then stained with PE-Cy7-CD11b (BD Pharmingen, United States), FITC-F4/80 (BD Pharmingen, United States) or PE-Ly6G (BD Pharmingen, United States). After staining, the cells were analyzed using the BD FACSVerse cytometer (BD Biosciences, United States). For each analysis, 50,000 events were acquired.

### Immunohistochemistry

The kidneys were fixed in 4% paraformaldehyde, followed by paraffin embedding and sectioning. Deparaffinized sections were sequentially stained with goat anti-mouse lymphatic vessel endothelial receptor 1 (Lyve-1) antibody (AF2125, R&D Systems, United States), biotin-conjugated rabbit anti-goat IgG (Solarbio, China) and horseradish peroxidase-streptavidin (Solarbio, China). The immunoreactivity was developed using diaminobenzidine (Vector Laboratories, United States). Images were acquired using the DM6000B microscope (Leica Microsystems, Germany). The number and positive area of lymphatic vessels around the interlobular arteries were quantified using ImageJ Pro (NIH, United States).

### Cell Culture

Primary HUVECs (ATCC, United States) were cultured in Vascular Cell Basal Medium supplemented with Endothelial Cell Growth Kit (ATCC, United States) at 37°C in a humidified atmosphere of 5% CO_2_. HUVECs from 2 to 8 passages were used for the indicated experiments. For the indicated transfections, HUVECs were seeded in 6-well plates and cultured for 24 h to reach 50–60% confluency. Then HUVECs were transfected with 40 nM agomir-505, 40 nM antagomir-505, 40 nM agomir-NC or 40 nM antagomir-NC using Lipofectamine RNAi MAX (Invitrogen, United States) following the manufacturer’s instructions. For LPS or TNF-α treatment, LPS (10 ng/mL) or TNF-α (2, 10 or 20 ng/mL) was added 24 h after the indicated transfections. THP-1 cells (ATCC, United States) were cultured in RPMI-1640 medium (Gibco, United States) supplemented with 10% fetal bovine serum (FBS) (Gibco, United States). For TNF-α stimulation, THP-1 cells were plated in 6-well plates and incubated with 10 ng/mL TNF-α for 24 h. Peripheral blood mononuclear cells (PBMCs) were isolated from C57/BL6 mice using Ficoll-Paque PREMIUM reagent (GE Healthcare, United States). PBMCs were cultured in RPMI-1640 medium supplemented with 10% FBS, followed by 10 ng/mL TNF-α stimulation for 24 h or 5 ng/mL LPS stimulation for 6 h.

### Real-Time Quantitative Polymerase Chain Reaction

Total RNA isolation was performed using TRIzol reagent (Invitrogen, United States) following the manufacturer’s instructions. MiRNA isolation was performed using miRNeasy Mini Kit (Qiagen, Germany) following the manufacturer’s protocols. Reverse transcription of total RNA and miRNA was performed using PrimeScript™ RT Master Mix (Takara, Japan) and miScript II RT Kit (Qiagen, Germany), respectively. Real-time PCR was performed using the Light Cycler 480 (Roche, Switzerland). RNU6B and GAPDH were included as the internal controls for the expression analyses of miR-505 and indicated genes, respectively. The primer sequences were indicated in the Table 1. Fold change of expression was calculated according to 2^–ΔΔCt^.

**TABLE 1:** Primer sequences.

Gene/miRNA name	Forward primer (5′–3′)	Reverse primer (5′–3′)
**Mouse**		
*Gapdh*	CCGGTGCTGAGTATGTCGTG	CCTTTTGGCTCCACCCTTC
*Il1b*	TGCCACCTTTTGACAGTGATG	AAGGTCCACGGGAAAGACAC
*Il6*	GCCTTCTTGGGACTGATGCT	TGCCATTGCACAACTCTTTTCT
*TNF-a*	ACGTCGTAGCAAACCACCAA	GCAGCCTTGTCCCTTGAAGA
*Icam1*	GGCACCCAGCAGAAGTTGTT	GCCTCCCAGCTCCAGGTATAT
*Vcam1*	GGAGAGACAAAGCAGAAGTGGAA	ACAACCGAATCCCCAACTTG
*E-selectin*	CCGTCCCTTGGTAGTTGCA	CAAGTAGAGCAATGAGGACGATGT
*Lyve1*	AATTTCACAGAAGCCAACGA	ATCCATAGCTGCAAGTCTC
**Human**		
*GAPDH*	CCCGCTCCCTCTTTCTTTG	GGGGCCATCCACAGTCTTC
*IL1B*	AACCTCTTCGAGGCACAAGG	GTCCTGGAAGGAGCACTTCAT
*IL6*	AAGCCAGAGCTGTGCAGATG	GCATTTGTGGTTGGGTCAGG
*TNFA*	CCCATGTTGTAGCAAACCCTC	TATCTCTCAGCTCCACGCCA
*ICAM1*	CTCCAATGTGCCAGGCTTG	CAGTGGGAAAGTGCCATCCT
*VACM1*	GAATGGGAGCTCTGTCACTGTAAG	ATTCAATCTCCAGCCGGTCA
*E-selectin*	CTGCCAAGTGGTAAAATGTTCAAG	CTTGCACACAGTGCCAAACAC
**miRNA**		
*RNU6B*	ACGCAAATTCGTGAAGCGTT	Universal primer
*hsa-miR-505*	CGTCAACACTTGCTGGTTTCCT	Universal primer
*mmu-miR-505*	CGTCAACACTTGCTGGTTTT	Universal primer

### Western Blotting

TNEN buffer (50 mM Tris–HCl 8.0, 150 mM NaCl, 5 mM EDTA and 1% NP-40) supplemented with protease inhibitors (Roche, United States) was used to lyse the cells. Cell lysate was run on 10% SDS-PAGE gel, followed by transfer and protein detection using rabbit monoclonal anti-vascular cell adhesion molecule 1 (VCAM1) antibody (ab134047, Abcam, United Kingdom). Anti-rabbit IgG antibody conjugated with alkaline phosphatase (Promega, United States) was used as the secondary antibody. NBT/BCIP substrate kit (Promega, United States) was used to visualize the protein band. The images of immunoblotting were acquired and quantified using the BioSpectrum Basic Imaging Systems (UVP, United States).

### Adhesion Assay

Cell adhesion was measured using a fluorometric method. In brief, HUVECs were seeded in 24-well plates and cultured to reach 50–60% confluency, followed by transfection of agomir-505, agomir-NC, antagomir-505 or antagomir-NC. Thirty-two hours later, HUVECs were treated with vehicle or 2 ng/mL TNF-α for 16 hr. THP-1 cells in the number of 2 × 10^5^ cells per well were then labeled with 5 μM CellTracker Green CMFDA (Thermo fisher, United States) in serum-free culture medium for 30 min, seeded on the monolayer of HUVECs and incubated for 1 hr. After incubation, the non-adherent THP-1 cells were washed off with PBS and the adherent cells were lysed in TNEN buffer supplemented with protease inhibitors. The fluorescence intensity was measured using a multi-mode microplate reader (BioTek Synergy 2, United States) with excitation/emission set at 485/535 nm.

### Human Subjects

Venous blood was collected from 52 normotensive healthy subjects and 31 previously untreated hypertensive patients from Yueyang Hospital, Shanghai University of Traditional Chinese Medicine. The patients were free of concomitant allergic and infectious conditions. Centrifugation was performed at 1,000 rpm for 10 min to isolate the plasma from the blood specimens. The plasma was stored at −80°C until the indicated analyses were performed. The clinical characteristics of the human subjects were summarized in the Table 2. The study complies with the Declaration of Helsinki and was approved by the Institutional Review Board of Yueyang Hospital of Integrated Traditional Chinese and Western Medicine, Shanghai University of Traditional Chinese Medicine (Approval No. 2013-011).

**TABLE 2:** Clinical characteristics of the human subjects.

Parameters	Normotensive subjects (*n* = 52; male = 36, female = 16)	Hypertensive patients (*n* = 31; male = 25, female = 6)
Age (years)	41.8 ± 1.46	46.8 ± 1.76
SBP (mmHg)	106.9 ± 1.34	144.3 ± 2.35*
DBP (mmHg)	67.1 ± 1.13	93.9 ± 1.68*
BMI (Kg/m^2^)	23.4 ± 0.37	26.32 ± 0.52*
Triglyceride (mmol/L)	1.32 ± 0.11	4.27 ± 1.43*
Cholesterol (mmol/L)	4.5 ± 0.14	4.83 ± 0.31
HDL (mmol/L)	1.29 ± 0.05	1.22 ± 0.05
LDL (mmol/L)	3.2 ± 0.16	2.90 ± 0.18

Data represent mean ± SEM. P values are derived from the Student’s t-test or Mann–Whitney U test. *Compared to that from normotensive subjects, p < 0.05.

### Enzyme-Linked Immunosorbent Assay

The level of soluble E-selectin (sE-selectin) was measured using Human E-selectin enzyme-linked immunosorbent assay (ELISA) kit (Multi Sciences, China) following the manufacturer’s instructions. The level of the plasma C-reactive protein (CRP) from human subjects was analyzed using a Human C-Reactive Protein/CRP Quantikine ELISA Kit (R&D, United States) following the manufacturer’s instructions. The absorbance was read at 450 nm and 570 nm using the Epoch Microplate Spectrophotometer (BioTek, United States).

### Statistical Analysis

All animal experiments and cell culture-based assays were performed with biological replicates and independently repeated at least three times. Data were presented as mean ± standard error of mean (S.E.M). Statistical comparisons were performed by unpaired Student’s *t* test or one-way analysis of variance (ANOVA), followed by Dunnett’s *post hoc* test. For the correlation analysis, non-parametric correlation (Spearman’s rho) was performed as the dependent variables were not normally distributed according to the normality tests (SPSS 16.0). The Mann–Whitney *U* test was used to compare the differences between the indicated groups. *P* value less than 0.05 was considered statistically significant.

## Results

### Hypertension May Result in Elevated Level of Plasma miR-505

**FIGURE 1:**
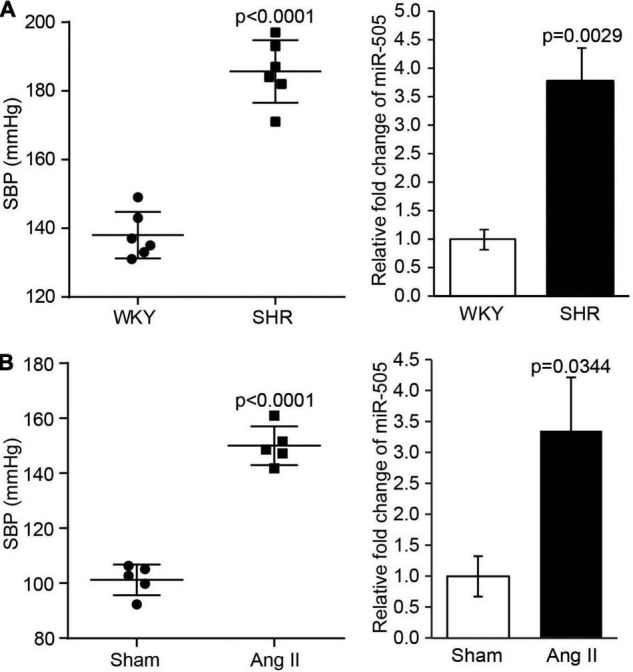
The level of plasma miR-505 is elevated in hypertensive animal models. **(A)** SBP was measured from 8-week-old SHRs and WKY rats (*n* = 6 per group). In addition, the level of plasma miR-505 from SHRs and WKY rats was analyzed by real-time PCR. Relative fold change of miR-505 was plotted against that from WKY controls. Data represent mean ± SEM. *P* values are derived from the Student’s *t*-test. **(B)** Eight-week-old C57BL/6J mice were subject to Ang II infusion at 1,000 ng/min/kg *via* osmotic mini-pumps for 2 weeks. Sham controls (Sham) received saline infusion in the same manner. SBP was measured at the end of the 2-week infusion (*n* = 5 per group). The plasma level of miR-505 from the Ang II-infused mice and sham controls was analyzed by real-time PCR. Relative fold change of miR-505 was plotted against that from the sham controls. Data represent mean ± SEM. *P* values are derived from the Student’s *t*-test.

Our previous study has revealed that the plasma level of miR-505 is elevated in hypertensive patients, which suggests the association between hypertension and elevation in the plasma miR-505 (4). To further understand whether a causal relationship exists between hypertension and elevated plasma level of miR-505, we examined the plasma level of miR-505 in SHRs, a genetically hypertensive model. As shown in Figure 1A, along with raised blood pressure, the plasma level of miR-505 was increased by approximately 3.8-fold in SHRs compared to that from the age- and sex-matched WKY controls. To further verify the causal relationship between hypertension and elevated plasma miR-505, pro-hypertensive Ang II was applied to induce the development of hypertension in mice, followed by analyzing the level of miR-505 in the plasma. As shown in Figure 1B, in addition to increases in the blood pressure, Ang II infusion resulted in elevated level of plasma miR-505 by about 3.4-fold compared to that from the vehicle-infused controls. These results indicate that hypertension may result in elevated level of miR-505 in the plasma.

### Elevated Level of Circulating miR-505 Leads to Increased Blood Pressure, Endothelial Dysfunction and Vascular Inflammation

**FIGURE 2:**
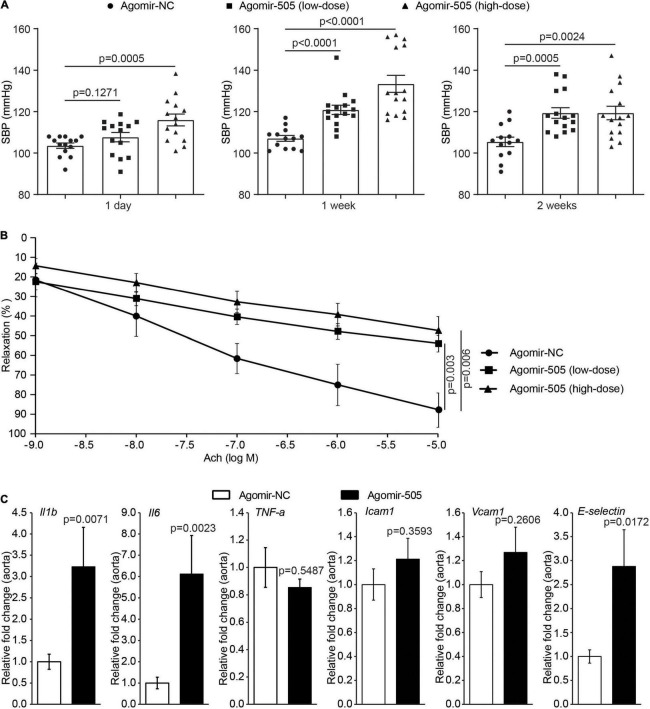
Elevated circulating miR-505 leads to raised blood pressure, impaired endothelium-dependent vasodilation and vascular inflammation. **(A)** C57/BL6 mice were treated with agomir-NC (10 mg/kg bw, *n* = 13) or agomir-505 (low-dose: 2.5 mg/kg bw, *n* = 15; high-dose: 10 mg/kg bw, *n* = 15) for 2 weeks through intravenous injection. SBP was measured 1 day, 1 week and 2 weeks after the termination of agomir-505 or agomir-NC administration. Data represent mean ± SEM. *P* values are derived from one-way ANOVA and the Student’s *t*-test. **(B)** Two weeks after the termination of agomir-505 or agomir-NC treatment, aortas were dissected and subject to the assessment of endothelium-dependent vasorelaxation to Ach. Data represent mean ± SEM. *P* values are derived from one-way ANOVA and the Student’s *t*-test. **(C)** Agomir-505 (10 mg/kg bw) or agomir-NC (10 mg/kg bw) was intravenously administered for 2 weeks. One week after the last injection, the aortas were dissected, followed by real-time quantitative polymerase chain reaction (qPCR) analyses to examine the expression of Il1b, Il6, TNF-a, Icam1, Vcam1 and E-selectin in agomir-505-treated (*n* = 4) or agomir-NC-treated mice (*n* = 7). Relative fold change of the expression of the indicated genes was plotted against that from agomir-NC-treated mice. Data represent mean ± SEM. *P* values are derived from the Student’s *t*-test.

To further elucidate the pathophysiological implications of increased circulating level of miR-505 in hypertension, agomir-NC or agomir-505 was intravenously administered to 6-week-old C57BL/6 mice at 2.5 mg/kg bw (low-dose) or 10 mg/kg bw (high-dose) and the blood pressure was subsequently monitored. As shown in Figure 2A, compared to agomir-NC-treated mice, SBP was increased in high-dose agomir-505-treated mice 1 day after agomir-505 treatment. By 1 week after the agomir-505 treatment, SBP was increased in both low-dose and high-dose agomir-505-treated mice. By 2 weeks after agomir-505 administration, SBP in low-dose and high-dose agomir-505-treated mice remained higher than that from the agomir-NC-treated mice. Next, the impact of miR-505 on endothelial function was assessed. Aortic rings dissected 2 weeks after agomir-505 or agomir-NC treatment were subject to vascular reactivity assessment. As shown in Figure 2B, compared to agomir-NC-treated mice, Ach-induced vasorelaxation was significantly impaired in PE-precontracted vessels isolated from both low-dose and high-dose agomir-505-treated mice. No significant changes in endothelium-dependent vasorelaxation were observed between low-dose and high-dose agomir-505 treatment. The vascular reactivity assay was also repeated 1 day and 1 week after agomir-505 treatment. Ach-induced vasorelaxation was markedly impaired in the aortic rings isolated from agomir-505-treated mice 1 day after agomir-505 treatment. Similar observations were also made 1 week after agomir-505 treatment (Supplementary Figure 1). Moreover, hypertension-associated endothelial dysfunction is characterized by attenuated endothelium-dependent vasorelaxation as well as endothelial inflammatory activation, which can cause impaired endothelium-dependent vasorelaxation (10). Thus, the aortic expression of proinflammatory genes including interleukin 1 beta (*Il1b*), interleukin 6 (*Il6*) and *TNF-*α as well as the markers of endothelial activation such as intercellular adhesion molecule 1 (*Icam1*), *Vcam1* and *E-selectin* was further analyzed. As shown in Figure 2C, the aortic expression of *Il1b*, *Il6* and *E-selectin* was increased by approximately 3.2, 6.1, and 2.9 fold, respectively, in agomir-505-treated mice compared to agomir-NC-treated mice. Taken together, these results indicate that hypertension *per se* causes increased level of plasma miR-505. On the other hand, elevated circulating miR-505 results in raised blood pressure, endothelial dysfunction and vascular inflammation.

### Elevated Level of Circulating miR-505 Results in Kidney Inflammation

**FIGURE 3:**
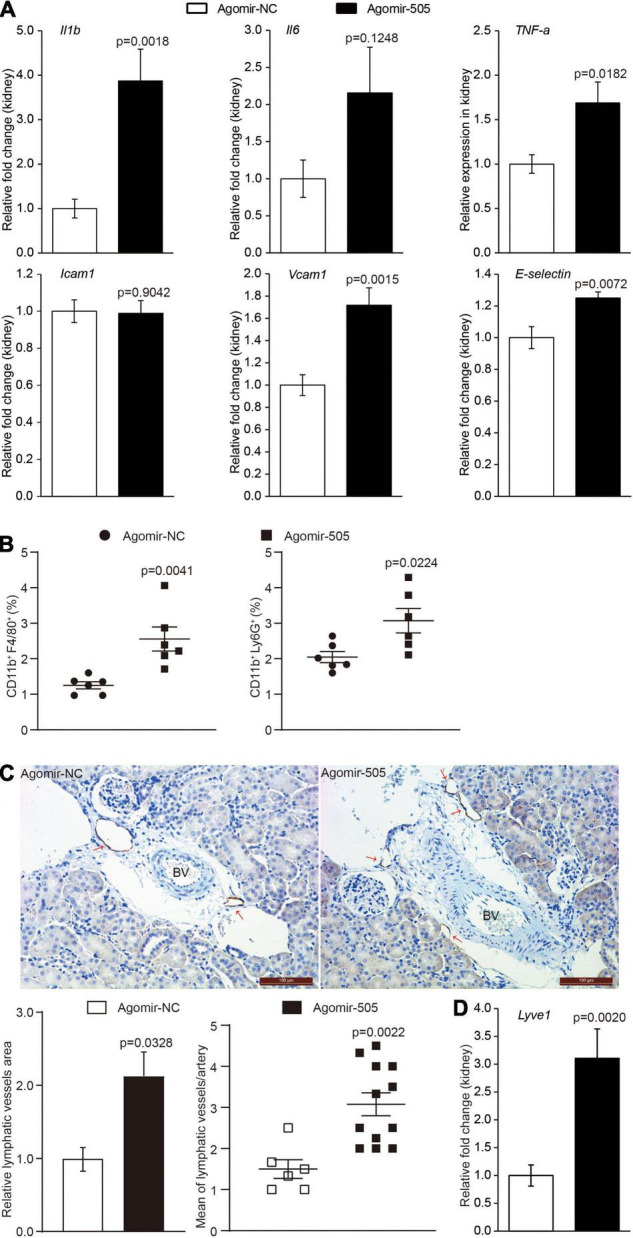
Elevated circulating level of miR-505 results in renal inflammation. Agomir-505 or agomir-NC was intravenously administered for 2 weeks. One week after the last injection, the kidneys were dissected, followed by gene expression or flowcytometry analyses. **(A)** Real-time qPCR analysis was performed to examine the renal expression of *Il1b*, *Il6*, *TNF-a*, *Icam1*, *Vcam1* and *E-selectin* in agomir-505-treated (*n* = 8) or agomir-NC-treated mice (*n* = 8). Relative fold change of the expression of the indicated genes was plotted against that from agomir-NC-treated mice. Data represent mean ± SEM. *P* values are derived from the Student’s *t*-test. **(B)** Flowcytometry analysis was performed to quantify the number of CD11b + F4/80 + macrophages or CD11b + Ly6G + neutrophils in the kidneys from agomir-505-treated (*n* = 6) or agomir-NC-treated mice (*n* = 6). Data represent mean ± SEM. *P* values are derived from the Student’s *t*-test. **(C)** Agomir-505 or agomir-NC was intravenously administered for 2 weeks. Two weeks later, the kidneys were dissected, followed by immunohistochemical examination and gene expression analyses. Immunohistochemistry was performed to visualize the lymphatic vessels in the kidneys from agomir-505-treated mice (*n* = 12) and agomir-NC-treated mice (*n* = 6). Lyve-1 positive area and the number of Lyve-1 positive lymphatic vessels (red arrows) around the interlobular arteries (BV) in the kidney were measured. BV, blood vessel. Scale bar: 100 μm. Data represent mean ± SEM. *P* values are derived from the Student’s *t*-test. **(D)** Real-time qPCR analysis was performed to analyze the renal expression of *Lyve-1* in agomir-505-treated mice (*n* = 12) and agomir-NC-treated mice (*n* = 6). Relative fold change of the expression of *Lyve-1* was plotted against that from agomir-NC-treated mice. Data represent mean ± SEM. *P* values are derived from the Student’s *t*-test.

To confirm the implication of miR-505 in hypertension-associated inflammation in target organs, the inflammatory changes in the kidney were also examined given that inflammatory kidney injuries occur as an early event during the development of hypertensive target organ damages. As shown in Figure 3A, the renal expression of *Il1b*, *TNF-*α, *Vcam1* and *E-selectin* was upregulated by approximately 3.9, 1.7-, 1.7- and 1.3-fold, respectively, in agomir-505-treated mice compared to agomir-NC-treated mice. To validate the inflammatory phenotype in the kidney, the number of CD11b^+^F4/80^+^ macrophages and CD11b^+^Ly6G^+^ neutrophils in the kidney was also analyzed by flowcytometry. The results showed that the number of CD11b^+^F4/80^+^ macrophages and that of CD11b^+^Ly6G^+^ neutrophils were increased in the kidneys from agomir-505-treated mice compared to agomir-NC-treated mice (Figure 3B). Furthermore, hypertension-associated renal inflammation is characterized and mediated by enhanced lymphangiogenesis (11, 12). Thus, immunohistochemical examination of lymphatic vessel endothelial receptor 1 (Lyve-1), a marker of lymphatic vasculature, was performed to visualize the lymphatic vessels in the kidney. As shown in Figure 3C, 1-2 lymphatic lumens were noted around the interlobular arteries in the kidney in agomir-NC-treated mice. In contrast, increased number of lymphatic lumens around the interlobular arteries and increased Lyve-1 positive area were noted in the kidneys from agomir-505-treated mice, demonstrating enhanced renal lymphangiogenesis as a result of agomir-505 treatment. Consistently, the renal expression of *Lyve-1* was increased by approximately 3.1-fold in agomir-505-treated mice compared to agomir-NC-treated mice (Figure 3D). Along with increased renal expression of *Il1b*, *TNF-*α, *Vcam1* and *E-selectin*, increased lymphatic vessel density and enhanced expression of *Lyve-1* in the kidney further confirm that elevated level of circulating miR-505 leads to kidney inflammation *in vivo*.

### MiR-505 Primes Immune Cells to Proinflammatory Responses

**FIGURE 4:**
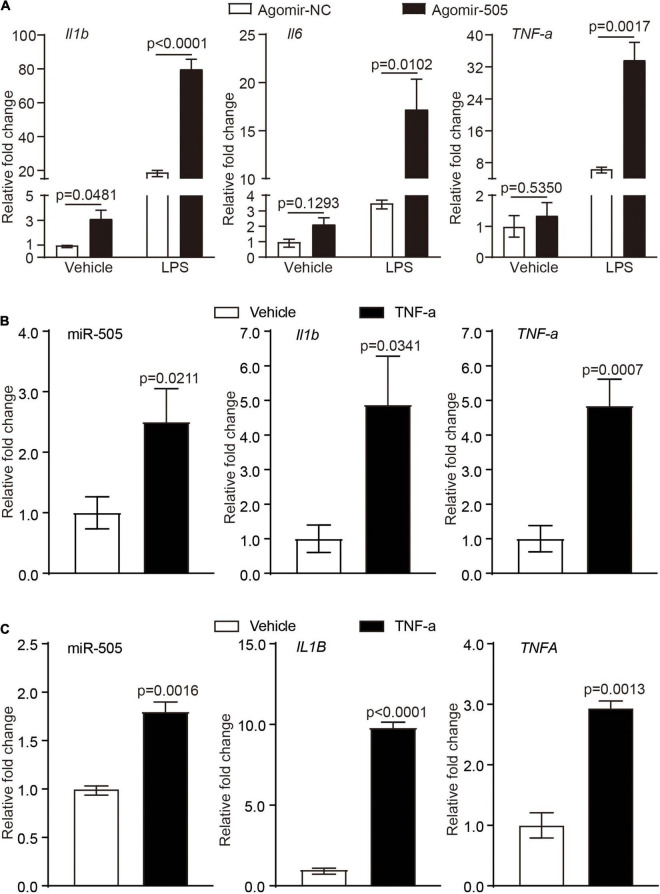
MiR-505 increases the sensitivity of immune cells to the proinflammatory stimuli. **(A)** Agomir-505 (10 mg/kg bw) or agomir-NC (10 mg/kg bw) was intravenously administered for 2 weeks. PMBCs isolated from agomir-505-treated (*n* = 5) and agomir-NC-treated mice (*n* = 3) were subject to vehicle or 5 ng/mL LPS stimulation for 6 h. The expression of *Il1b*, *Il6* and *TNF-*α was then analyzed by real-time qPCR analysis. Relative fold change was plotted against that from agomir-NC and vehicle-treated PBMCs. Data represent mean ± SEM. *P* values are derived from one-way ANOVA and the Student’s *t*-test. **(B)** PBMCs isolated from C57/BL6 mice were treated by vehicle or 10 ng/mL TNF-α for 24 h, followed by real-time qPCR analysis of the expression of miR-505, *Il1b* and *TNF-*α. Relative fold change was plotted against that from vehicle-treated PBMCs (*n* = 4 per group). Data represent mean ± SEM. *P* values are derived from the Student’s *t*-test. **(C)** THP-1 cells were treated by vehicle or 10 ng/mL TNF-α for 24 h, followed by real-time qPCR analysis of the expression of miR-505, IL1B and TNFA. Relative fold change was plotted against that from vehicle-treated THP-1 cells (*n* = 4 per group). Data represent mean ± SEM. *P* values are derived from the Student’s *t*-test.

Activated immune cells are critical players in mediating hypertension-associated inflammatory injuries in the target organs. Given that agomir-505 *in vivo* delivery resulted in inflammatory changes in the kidney, the implication of miR-505 in immune cell-mediated inflammatory responses was further investigated. First, peripheral blood mononuclear cells (PBMCs) were collected from the mice treated with agomir-505 or agomir-NC, followed by the expression analysis of *Il1b*, *Il6* and *TNF-*α in the absence or presence of LPS stimulation. As shown in Figure 4A, compared to agomir-NC-treated mice, the mRNA level of *Il1b* was increased by about 3.1-fold in PBMCs in the absence of LPS stimulation. Moreover, LPS-stimulated expression of *Il1b*, *Il6* and *TNF-*α was further increased in PBMCs from agomir-505-treated mice compared to agomir-NC-treated PBMCs. Next, we examined the expression of miR-505 in response to TNF-α stimulation in PBMCs. As shown in Figure 4B, the expression of miR-505 was upregulated by approximately 2.5-fold in TNF-α-stimulated PBMCs compared to vehicle-treated cells. Meanwhile, as a result of TNF-α stimulation, the mRNA levels of *Il1b* and *TNF-*α were increased by approximately 4.9- and 4.8-fold, respectively, verifying an inflammatory state of the cells. Similar observations were also made in THP-1 cells, a human monocyte cell line. The expression of miR-505 was increased by about 1.8-fold in TNF-α-stimulated THP-1 cells (Figure 4C). In the meantime, the expression of *IL1B* and *TNFA* was increased by approximately 9.8 and 2.9 fold, respectively. Taken together, these results indicate that proinflammatory mediators such as TNF-α can upregulate the level of miR-505 in immune cells. Meanwhile, elevated circulating level of miR-505 may result in increased susceptibility of mononuclear immune cells to proinflammatory stimuli-induced inflammatory responses.

### MiR-505 Directly Promotes Endothelial Inflammation and Activation

**FIGURE 5:**
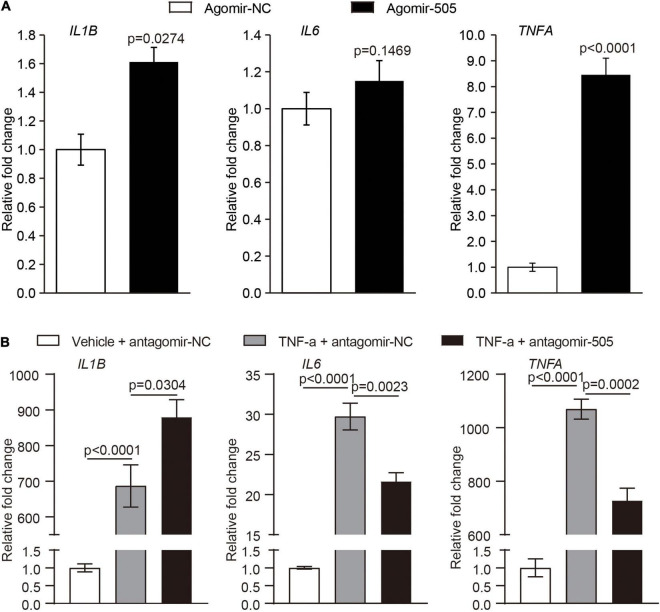
MiR-505 upregulates the expression of proinflammatory genes in endothelial cells. **(A)** Agomir-505 or agomir-NC was transfected in HUVECs, followed by real-time qPCR analysis of the expression of *IL1B*, *IL6* and *TNFA*. Relative fold change of gene expression was plotted against that from agomir-NC-transfected HUVECs (*n* = 6 per group). Data represent mean ± SEM. *P* values are derived from the Student’s *t*-test. **(B)** Antagomir-505 or antagomir-NC was transfected in HUVECs, followed by 2 ng/mL TNF-α stimulation for 16 h. The expression of *IL1B*, *IL6* and *TNFA* was then examined by real-time qPCR analysis. The relative fold change of gene expression was plotted against that from antagomir-NC-transfected HUVECs in the absence of TNF-α stimulation (vehicle + antagomir-NC) (*n* = 6 per group). Data represent mean ± SEM. *P* values are derived from one-way ANOVA and the Student’s *t*-test.

Given that endothelial dysfunction and increased aortic expression of proinflammatory genes were observed as a result of agomir-505 administration *in vivo*, the direct implication of miR-505 in endothelial inflammation was further investigated. The expression of *IL1B*, *IL6* and *TNFA* was analyzed in HUVECs transfected with agomir-505 or agomir-NC. As shown in Figure 5A, compared to agomir-NC-transfected HUVECs, the mRNA levels of *IL1B* and *TNFA* were upregulated by approximately 1.6- and 8.5-fold, respectively, in agomir-505-transfected HUVECs. Next, the expression of miR-505 in response to TNF-α stimulation was examined in endothelial cells. No significant changes in the level of miR-505 were observed in TNF-α-stimulated HUVECs (Supplementary Figure 2). These observations suggest that elevated level of miR-505 promotes the expression of proinflammatory genes such as *IL1B* and *TNFA* in HUVECs. However, miR-505 itself is not under the regulation of TNF-α in HUVECs.

To further examine the possibility that miR-505 is required for endothelial inflammation, antagomir-505 was applied to suppress the activity of the endogenous miR-505. Given that the basal level of proinflammatory gene expression is low under normal conditions, TNF-α was applied to induce the expression of proinflammatory genes in HUVECs. As shown in Figure 5B, compared to antagomir-NC-transfected TNF-α-stimulated HUVECs, TNF-α-induced expression of *IL6* and *TNFA* was significantly attenuated in antagomir-505-transfected HUVECs. Similar observations were made when HUVECs were subject to lipopolysaccharide (LPS) stimulation. In response to LPS stimulation, antagomir-505 transfection resulted in significantly reduced expression of *IL1B*, *IL6* and *TNFA* compared to antagomir-NC-transfected LPS-stimulated HUVECs (Supplementary Figure 3). Taken together, these results indicate that miR-505 promotes the inflammatory responses in HUVECs.

**FIGURE 6:**
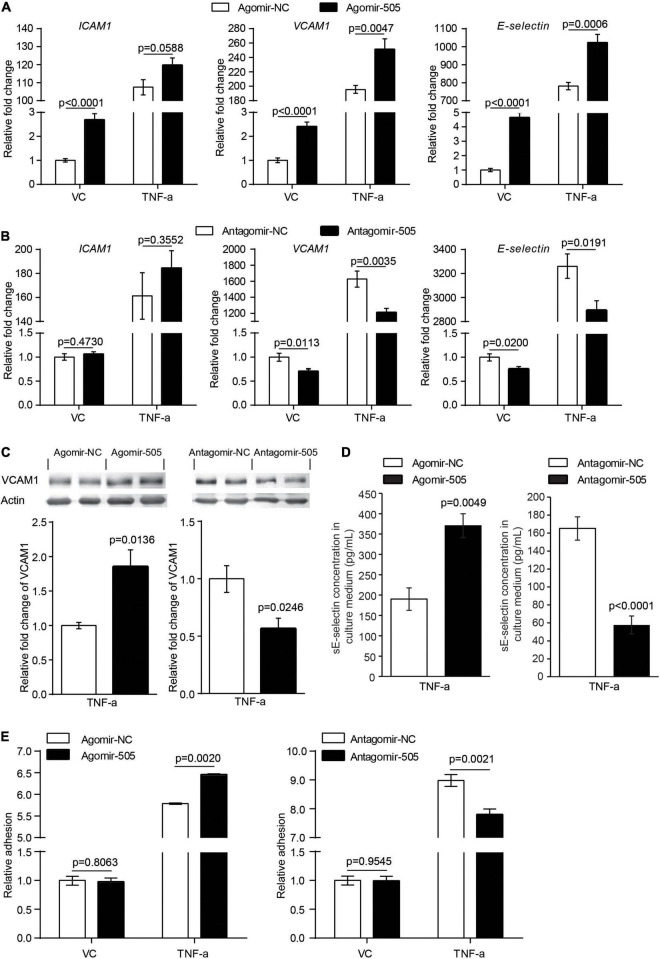
MiR-505 promotes endothelial activation. **(A)** Agomir-505-transfected and agomir-NC-transfected HUVECs were exposed to vehicle (VC) or 2 ng/mL TNF-α for 16 h, followed by real-time qPCR analysis of the expression of *ICAM1*, *VCAM1* and *E-selectin*. Relative fold change of gene expression was plotted against that from agomir-NC-transfected vehicle-treated cells (*n* = 6 per group). Data represent mean ± SEM. *P* values are derived from one-way ANOVA and the Student’s *t*-test. **(B)** Antagomir-505-transfected and antagomir-NC-transfected HUVECs were treated with vehicle or 2 ng/mL TNF-α for 16 h, followed by real-time qPCR analysis of the expression of *ICAM1*, *VCAM1* and *E-selectin*. Relative fold change of gene expression was plotted against that from antagomir-NC-transfected vehicle-treated cells (*n* = 6 per group). Data represent mean ± SEM. *P* values are derived from one-way ANOVA and the Student’s *t*-test. **(C)** Agomir-505, antagomir-505 and their respective negative controls were transfected in HUVECs, followed by vehicle or 2 ng/mL TNF-α treatment for additional 16 h. The protein level of VCAM1 was then examined by western blotting. Relative fold change of VCAM1 protein was plotted against that from agomir-NC-transfected TNF-α-stimulated cells or antagomir-NC-transfected TNF-α-stimulated cells (*n* = 4 per group). Data represent mean ± SEM. *P* values are derived from the Student’s *t*-test. **(D)** HUVECs were transfected with agomir-505, antagomir-505 and their respective negative controls, followed by vehicle or 2 ng/mL TNF-α treatment for 12 h. The culture medium was then collected and analyzed for the level of sE-selectin by ELISA (*n* = 6 per group). Data represent mean ± SEM. *P* values are derived from the Student’s *t*-test. **(E)** Agomir-505, antagomir-505 and their respective negative controls were transfected in HUVECs, followed by vehicle or 2 ng/mL TNF-α treatment for additional 16 h. The adhesion of CMFDA-labeled THP-1 cells to HUVECs was then examined by measuring the fluorescence intensity of CMFDA. Relative fold change of adhesion was plotted against that from agomir-NC-transfected vehicle-treated cells or antagomir-505-transfected vehicle-treated cells (*n* = 6 per group). Data represent mean ± SEM. *P* values are derived from one-way ANOVA and the Student’s *t*-test.

Moreover, given that inflammation causes endothelial activation, the impact of miR-505 on the expression of endothelial activation mediators *ICAM1*, *VCAM1* and *E-selectin* was analyzed in the absence or presence of TNF-α stimulation. As shown in Figure 6A, without TNF-α stimulation, the mRNA levels of *ICAM1*, *VCAM1* and *E-selectin* were increased by approximately 2.7-, 2.4- and 4.7-fold as a result of agomir-505 transfection. In the presence of TNF-α, agomir-505-transfected HUVECs exhibited significantly higher levels of *VCAM1* and *E-selectin* compared to agomir-NC-transfected HUVECs. On the other hand, antagomir-505-transfected HUVECs were characterized by decreased expression of *VCAM1* and *E-selectin* compared to antagomir-NC-transfected HUVECs. Meanwhile, TNF-α-induced expression of *VCAM1* and *E-selectin* was significantly attenuated in antagomir-505-transfected cells (Figure 6B). The impact of miR-505 on the expression of VCAM1 and E-selectin was also validated at the protein level. In the presence of TNF-α stimulation, increased protein level of VCAM1 was observed in agomir-505-transfected HUVECs compared to agomir-NC-transfected cells. However, compared to antagomir-NC-transfected TNF-α-stimulated cells, the protein level of VCAM1 was significantly decreased in antagomir-505-transfected TNF-α-stimulated cells (Figure 6C). The level of soluble E-selectin (sE-selectin) was also modulated by miR-505. As shown in Figure 6D, in the presence of TNF-α stimulation, agomir-505 transfection resulted in elevated level of sE-selectin. On the contrary, the level of sE-selectin was significantly decreased in antagomir-505-transfected cells.

Lastly, at the cellular level, endothelial inflammatory activation is characterized by increased adhesion of leukocytes to the activated endothelium. To evaluate the cellular effect of miR-505 on endothelial inflammatory activation, agomir-505 or antagomir-505 was transfected in HUVECs and stimulated by TNF-α. The adhesion of THP-1 cells to HUVECs was then examined. As shown in Figure 6E, compared to agomir-NC-transfected HUVECs, agomir-505 transfection resulted in approximately 12% increase in the adhesion of THP-1 cells to TNF-α-stimulated HUVECs, whereas antagomir-505 transfection led to about 13% decrease in the adhesion of THP-1 cells to TNF-α-stimulated HUVECs. Collectively, these results support that miR-505 may enhance or synergize with proinflammatory mediators to promote endothelial inflammatory activation.

### The Plasma Level of miR-505 Is Correlated With the Blood Pressure and the Level of CRP in Human Subjects

**FIGURE 7:**
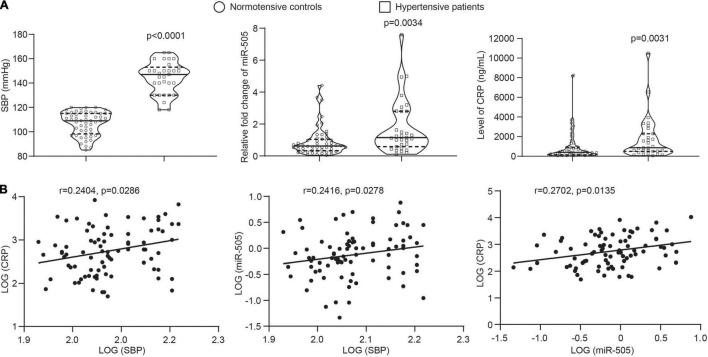
The plasma level of miR-505 is positively correlated with CRP and SBP. **(A)** The plasma levels of CRP and miR-505 were analyzed in 52 normotensive subjects and 31 untreated hypertensive patients by ELISA and real-time qPCR, respectively. Data represent medians with interquartile range. *P* values are derived from Mann–Whitney *U* test. **(B)** Correlation between the plasma level of CRP and SBP, the plasma level of miR-505 and SBP, and the plasma levels of CRP and miR-505 were determined with Spearman’s rho.

The results shown above support a direct role of miR-505 in promoting inflammatory gene expression in endothelial cells and immune cells as well as its function in augmenting inflammatory responses in the context of hypertension. To further assess the clinical relevance of miR-505 in hypertension-associated inflammation, the circulating level of miR-505 was examined in an independent cohort of previously untreated hypertensive patients along with that of CRP, a well-established biomarker of inflammation closely associated with the development of hypertension and endothelial dysfunction (13). Consistent with our previous report (4), the plasma level of miR-505 was increased in the hypertensive patients compared to the normotensive subjects. Meanwhile, elevated plasma CRP was also observed in the hypertensive patients (Figure 7A). Correlation analyses revealed that SBP readings were positively correlated with the level of CRP or miR-505. Meanwhile, a positive correlation of the plasma level of CRP with the level of miR-505 was also noted (Figure 7B). These results not only verify that the plasma levels of miR-505 and CRP are elevated in hypertensive patients, but also suggest that the plasma level of miR-505 is positively associated with the inflammatory status under hypertensive conditions.

## Discussion

Hypertension is not only a vascular disorder, but also an inflammatory condition. Endothelial dysfunction is a major feature of hypertension-associated vascular pathologies. Inflammation plays a central role in the development of hypertensive target organ damages including endothelial dysfunction. Understanding the pathophysiological implications of circulating miRNAs in hypertension-associated vascular and inflammatory alterations may offer new opportunities for the timely detection and precise prognostication of hypertensive target organ damages. As a continued study from our previous work that has identified elevated plasma level of miR-505 in hypertensive patients (4), the current study further reveals that not only hypertension *per se* causes elevated level of miR-505 in the plasma, elevated plasma miR-505 may also result in raised blood pressure, endothelial dysfunction, vascular inflammation, kidney inflammation and pre-activation of mononuclear immune cells *in vivo*. In addition, the work here uncovers that miR-505 promotes endothelial inflammatory activation in a cell-autonomous manner, which may in part explain miR-505-mediated endothelial dysfunction *in vivo*. Furthermore, the current work demonstrates that the plasma level of miR-505 is positively correlated with SBP and the level of CRP in human subjects, lending addition support to the potential clinical implication of miR-505 in hypertension-associated inflammation.

First, in addition to demonstrating the casual relationship between hypertension and elevated level of miR-505 in the plasma, the work here presents experimental evidence supporting the implication of miR-505 in the development of hypertension and endothelial dysfunction. Hypertension and endothelial dysfunction mutually affect and reinforce each other in promoting the pathological development of cardiovascular complications (14, 15). Hypertension can cause endothelial dysfunction. Meanwhile, endothelial dysfunction may accelerate and aggravate hypertension. Mechanism-based assessment of endothelial dysfunction may help improve the early detection and prognostication of hypertension-associated cardiovascular complications. Our previous study has reported that the plasma level of miR-505 is elevated in hypertensive patients, suggesting the association between hypertension and elevation in the plasma miR-505. We have also observed that miR-505 impairs the migration and capillary tube formation in endothelial cells (4). However, it remains unknown whether hypertension results in elevated level miR-505 in the plasma and elevated miR-505 in circulation causes endothelial dysfunction *in vivo*. Utilizing both genetically hypertensive SHRs and Ang II-induced hypertensive models, we demonstrate that elevated plasma level of miR-505 is a result of hypertension (Figure 1), supporting a causal relationship between hypertension and the elevation of miR-505 in the plasma. Moreover, the current work shows that intravenous delivery of agomir-505 raises the blood pressure in mice, providing direct evidence supporting the pro-hypertensive function of miR-505 *in vivo* (Figure 2A). Most notably, agomir-505 *in vivo* administration results in significant impairment of endothelium-dependent vasorelaxation (Figure 2B). The significantly impaired endothelium-dependent vasorelaxation is noted as early as 1 day after agomir-505 treatment (Supplementary Figure 1), suggesting a direct impact of miR-505 on endothelial dysfunction. Taken together, the work here suggests that elevated plasma miR-505 may in part contribute to the development of endothelial dysfunction under hypertensive conditions. In addition, our previous work has shown that nuclear protein high mobility group box 1 (HMGB1) is a predicted target of miR-505. Meanwhile, miR-505 suppresses the expression of HMGB1 in HUVECs (4). Of interest, a recent study has demonstrated that endothelial cell-specific knockout of HMGB1 results in elevated blood pressure and impaired endothelium-dependent vasorelaxation in mice (16). Thus, it is likely that HMGB1 is one of the direct targets of miR-505 in regulating blood pressure and endothelial function *in vivo*. Future studies are necessary to further test this hypothesis, validating the targeted regulation of HMGB1 by miR-505 in relation to its implications in hypertension and endothelial dysfunction. Furthermore, although the work here presents experimental evidence supporting the role of miR-505 in endothelial dysfunction *in vivo* (Figure 2A and Supplementary Figure 1), no significant changes in the levels of nitric oxide and oxidative stress were observed in agomir-505-transfected HUVECs (data not shown). One possibility is that miR-505 may act in synergy with other factors in promoting endothelial dysfunction. If any, the cell-autonomous effects of miR-505 on the production of nitric oxide and reactive oxygen species in endothelial cells remain to be elucidated in future studies. In the meantime, it should be noted that our *in vivo* experiments were carried out in male animals, the findings of the current work are thus not applicable to female subjects without further validations in female models.

Second, in addition to endothelial dysfunction, our work here suggests the implication of miR-505 in hypertension-associated inflammation. Agomir-505 treatment results in vascular inflammation (Figure 2C), kidney inflammation (Figure 3) and enhanced inflammatory responses in PBMCs (Figure 4A) *in vivo*, linking the level of miR-505 to inflammatory changes in hypertensive target organs *in vivo*. It has been noted that PBMCs from hypertensive patients manifest increased susceptibility to inflammatory activation (17). Our findings therefore suggest that elevated plasma miR-505 may contribute to pre-activation of immune cells in hypertensive patients. Meanwhile, a direct role of miR-505 in promoting endothelial inflammatory activation is further demonstrated (Figures 5, 6). Agomir-505 transfection in HUVECs results in elevated expression of proinflammatory *Il1B* and *TNFA* as well as vascular adhesion molecules *ICAM1*, *VCAM1* and *E-selectin*, suggesting that miR-505 directly promotes endothelial inflammatory activation. MiR-505 also augments TNF-α-mediated induction of *VCAM1* and *E-selectin* in endothelial cells, implying a synergy between elevated level of miR-505 and inflammatory mediators in inducing endothelial inflammatory activation. Endothelial activation facilitates the adhesion of circulating leukocytes to the endothelium, a phenomenon noted in hypertension and contributes to the development of atherosclerotic pathologies (18). Indeed, the endothelial level of miR-505 affects the interaction of monocytes and endothelial cells. Agomir-505 transfection in HUVECs results in increased adhesion of THP-1 cells, whereas antagomir-505 reduces the adhesion of THP-1 cells to HUVECs, verifying at the cellular level that miR-505 promotes endothelial activation. The vascular adhesion molecules such as *ICAM1*, *VCAM1* and *E-selectin* are normally expressed at barely detectable levels on the surface of the vascular endothelium. The expression of these genes is typically induced by proinflammatory cytokines such as TNF-α and IL6. Thus, it is likely that elevated expression of *ICAM-1*, *VCAM-1* and *E-selectin* in agomir-505-transfected HUVECs may in part result from miR-505-induced expression of TNF-α. Additional studies are required to elucidate the mechanisms involved in miR-505-mediated regulation of *Il1B* and *TNFA* as well as E-selectin and VCAM-1 in endothelial cells. Nevertheless, given that endothelial dysfunction is characterized by impaired endothelium-dependent vasodilation and endothelial inflammatory activation, the direct role of miR-505 in mediating endothelial inflammatory activation lends additional support to the prognostic value of circulating miR-505 as a biomarker for hypertension-associated endothelial dysfunction. Meanwhile, endothelial dysfunction constitutes the first step in the development of atherosclerosis and predicts future cardiac events. Upregulation of endothelial adhesion molecules such as E-selectin and VCAM-1 represents the early event initiating atherogenesis (19). Thus, the findings here also warrant future studies to further investigate the potential association of circulating miR-505 with atherosclerosis in hypertensive patients. Moreover, it is worth noting that the expression of miR-505 in endothelial cells does not seem be affected by proinflammatory TNF-α (Supplementary Figure 2) although miR-505 promotes endothelial inflammation. Instead, the level of miR-505 is significantly increased in TNF-α-stimulated PBMCs as well as THP-1 cells (Figures 4B,C), suggesting that the level of miR-505 is subject to the regulation of proinflammatory factors such as TNF-α in immune cells.

The impact of miR-505 on the inflammatory responses in endothelial and immune cells is closely relevant to hypertension-associated pathophysiological changes in target organs. During the course of hypertension, the interaction between immune cells and endothelial cells plays a critical role in initiating and propagating the inflammatory insults in the target organs. Activated immune cell-mediated inflammation plays an important role in the pathogenesis of hypertension and hypertensive target organ damages. For instance, monocytes secrete proinflammatory factors, trigger oxidative stress, express coagulating factors and transform into macrophages, thereby initiating vascular inflammation and hypertensive target organ damages such as kidney damage (20). Moreover, inflammation-triggered upregulation of adhesion molecules in endothelial cells further promotes the infiltration and accumulation of monocytes into target organs, exacerbating target organ damages. TNF-α is one of the most important inflammatory mediators in this process (21). The peripheral blood monocytes from hypertensive patients are marked by increased expression and secretion of TNF-α upon proinflammatory stimulation (17). The plasma level of TNF-α is increased in hypertensive patients and independently predicts coronary endothelial dysfunction in hypertensive patients (22, 23). Meanwhile, TNF-α contributes to hypertensive target organ damages including kidney injuries (24). The newly identified action of miR-505 in augmenting the level of proinflammatory mediators such as TNF-α in PBMCs (Figure 4A) corroborates vascular inflammation (Figure 2C) and kidney inflammation (Figure 3) as a result of agomir-505 *in vivo* administration. Given that monocytes and endothelial cells are major cellular bases of inflammatory responses in hypertension, our work here highlights a novel role of miR-505 in the pathogenesis of hypertension-associated inflammation. Meanwhile, the results here also provide a new clue to further elucidate the mechanisms regulating the expression of TNF-α under hypertensive conditions. Future studies are necessary to elucidate how TNF-α is regulated by miR-505 in endothelial cells and how TNF-α regulates the expression of miR-505 in immune cells. Additional studies are also required to elucidate if miR-505 mediates the effects of TNF-α in immune cells or acts independently instead.

The prognostic value of miR-505 in hypertension-associated inflammation is further supported by the clinical findings showing that the plasma level of miR-505 is positively correlated with that of CRP as well as SBP (Figure 7). As a marker of systemic inflammation, the level of CRP is associated with the development of hypertensive target organ damages including vascular alterations and cardiovascular events in hypertensive patients (13). Consistently, our work reveals that the level of CRP was elevated and positively correlated with SBP, corroborating the notion that hypertension is an inflammatory condition. Meanwhile, the plasma level of miR-505 was positively correlated with the blood pressure readings as well as the level of CRP. The positive correlation of plasma miR-505 with CRP is consistent with the above-mentioned pre-clinical findings of the pro-inflammatory function of miR-505, further supporting that miR-505 is associated with systemic inflammation in hypertension. Future studies are worth pursuing to assess the clinical significance of the combination of miR-505 and CRP in prognosticating inflammation and target organ impairment in hypertensive patients.

## Conclusion

**FIGURE 8:**
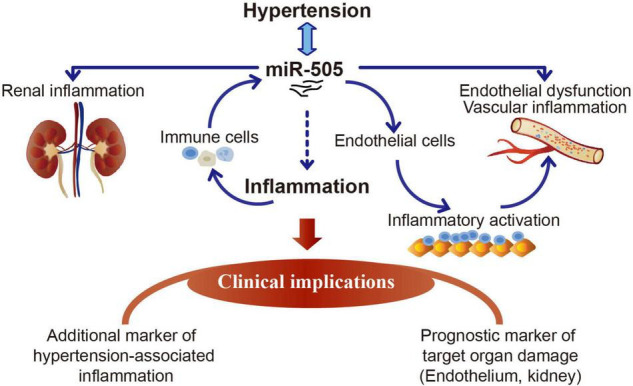
A schematic drawing of the potential clinical implications of miR-505 in hypertension. Elevated circulating miR-505 leads to raised blood pressure, endothelial dysfunction, vascular inflammation, kidney inflammation and pre-activation of peripheral mononuclear immune cells. The functional involvement of miR-505 in inflammation may partially explain its potential implications in hypertension-associated endothelial dysfunction and inflammation in target organs such as the kidney. MiR-505 can be upregulated by pro-inflammatory stimuli in mononuclear immune cells. Meanwhile, miR-505 augments the inflammatory response in mononuclear immune cells, which may promote the inflammatory injuries in target organs. In addition, miR-505 mediates endothelial inflammatory activation in a cell-autonomous manner, which may cause endothelial dysfunction and contribute in part to blood pressure elevation.

The work here demonstrates that elevated plasma miR-505 is not only a result of hypertension, but also promotes the development of hypertension, hypertensive endothelial dysfunction, kidney inflammation and pre-activation of immune cells. The functional implications of miR-505 in the proinflammatory responses in immune cells and endothelial cells have also been demonstrated in this study. MiR-505 is upregulated by TNF-α in THP-1 cells or PBMCs and functions to promote endothelial inflammation in a cell-autonomous manner. The findings here thus warrant further evaluation of miR-505 as a new prognostic biomarker for the early detection and risk prediction of hypertension-associated endothelial dysfunction and inflammation (Figure 8). Future studies are required to elucidate the direct gene targets that mediate miR-505-induced endothelial dysfunction and inflammation. The therapeutic potentials of miR-505-targeted treatment during the course of hypertension are also worth pursuing in the future.

## Data Availability Statement

The original contributions presented in the study are included in the article/Supplementary Material, further inquiries can be directed to the corresponding authors.

## Ethics Statement

The studies involving human participants were reviewed and approved by the Institutional Review Board of Yueyang Hospital of Integrated Traditional Chinese and Western Medicine, Shanghai University of Traditional Chinese Medicine. Written informed consent for participation was not required for this study in accordance with the national legislation and the institutional requirements. The animal study was reviewed and approved by the Institutional Animal Care and Use Committee at Yueyang Hospital of Integrated Traditional Chinese and Western Medicine, Shanghai University of Traditional Chinese Medicine.

## Author Contributions

TZ and YC oversaw the project. QY, PW, YQC, YMC, JC, and XD carried out the experiments. QY, PW, YQC, and YMC analyzed the data. YC and QY drafted the manuscript. YC and TZ revised the manuscript. All authors contributed to the article and approved the submitted version.

## Conflict of Interest

The authors declare that the research was conducted in the absence of any commercial or financial relationships that could be construed as a potential conflict of interest.

## Publisher’s Note

All claims expressed in this article are solely those of the authors and do not necessarily represent those of their affiliated organizations, or those of the publisher, the editors and the reviewers. Any product that may be evaluated in this article, or claim that may be made by its manufacturer, is not guaranteed or endorsed by the publisher.

## References

[1] Panza JA Quyyumi AA Brush JE Jr. Epstein SE. Abnormal endothelium-dependent vascular relaxation in patients with essential hypertension. *N Engl J Med.* (1990) 323:22–7. 10.1056/nejm199007053230105 2355955

[2] Savoia C Schiffrin EL. Inflammation in hypertension. *Curr Opin Nephrol Hypertens.* (2006) 15:152–8. 16481882 10.1097/01.mnh.0000203189.57513.76

[3] Androulakis ES Tousoulis D Papageorgiou N Tsioufis C Kallikazaros I Stefanadis C. Essential hypertension: is there a role for inflammatory mechanisms? *Cardiol Rev.* (2009) 17:216–21. 10.1097/CRD.0b013e3181b18e03 19690472

[4] Yang Q Jia C Wang P Xiong M Cui J Li L MicroRNA-505 identified from patients with essential hypertension impairs endothelial cell migration and tube formation. *Int J Cardiol.* (2014) 177:925–34. 10.1016/j.ijcard.2014.09.204 25449503

[5] Escate R Mata P Cepeda JM Padreó T Badimon L. miR-505-3p controls chemokine receptor up-regulation in macrophages: role in familial hypercholesterolemia. *FASEB J.* (2018) 32:601–12. 10.1096/fj.201700476r 32172543

[6] He L Hannon GJ. MicroRNAs: small RNAs with a big role in gene regulation. *Nat Rev Genet.* (2004) 5:522–31. 10.1038/nrg1379 15211354

[7] Navickas R Gal D Laucevièius A Taparauskaitë A Zdanytë M Holvoet P. Identifying circulating microRNAs as biomarkers of cardiovascular disease: a systematic review. *Cardiovasc Res.* (2016) 111:322–37. 10.1093/cvr/cvw174 27357636 PMC4996262

[8] Creemers EE Tijsen AJ Pinto YM. Circulating microRNAs: novel biomarkers and extracellular communicators in cardiovascular disease? *Circ Res.* (2012) 110:483–95. 10.1161/CIRCRESAHA.111.247452 22302755

[9] D’Alessandra Y Devanna P Limana F Straino S Di Carlo A Brambilla PG Circulating microRNAs are new and sensitive biomarkers of myocardial infarction. *Eur Heart J.* (2010) 31:2765–73. 10.1093/eurheartj/ehq167 20534597 PMC2980809

[10] Watson T Goon PK Lip GY. Endothelial progenitor cells, endothelial dysfunction, inflammation, and oxidative stress in hypertension. *Antioxid Redox Signal.* (2008) 10:1079–88. 10.1089/ars.2007.1998 18315493

[11] Kneedler SC Phillips LE Hudson KR Beckman KM Lopez Gelston CA Rutkowski JM Renal inflammation and injury are associated with lymphangiogenesis in hypertension. *Am J Physiol Renal Physiol.* (2017) 312:F861–9. 10.1152/ajprenal.00679.2016 28228406 PMC5451556

[12] Pei G Yao Y Yang Q Wang M Wang Y Wu J Lymphangiogenesis in kidney and lymph node mediates renal inflammation and fibrosis. *Sci Adv.* (2019) 5:eaaw5075. 10.1126/sciadv.aaw5075 31249871 PMC6594767

[13] Hage FG. C-reactive protein and hypertension. *J Hum Hypertens.* (2014) 28:410–5. 10.1038/jhh.2013.111 24226100

[14] Brandes RP. Endothelial dysfunction and hypertension. *Hypertension.* (2014) 64:924–8. 10.1161/hypertensionaha.114.03575 25156167

[15] Taddei S Virdis A Mattei P Ghiadoni L Fasolo CB Sudano I Hypertension causes premature aging of endothelial function in humans. *Hypertension.* (1997) 29:736–43. 10.1161/01.hyp.29.3.736 9052889

[16] Zhou Q Tu T Tai S Tang L Yang H Zhu Z. Endothelial specific deletion of HMGB1 increases blood pressure and retards ischemia recovery through eNOS and ROS pathway in mice. *Redox Biol.* (2021) 41:101890. 10.1016/j.redox.2021.101890 33582562 PMC7887649

[17] Dörffel Y Lätsch C Stuhlmüller B Schreiber S Scholze S Burmester GR Preactivated peripheral blood monocytes in patients with essential hypertension. *Hypertension.* (1999) 34:113–7. 10.1161/01.hyp.34.1.113 10406833

[18] McCarron RM Wang L Sirén AL Spatz M Hallenbeck JM. Monocyte adhesion to cerebromicrovascular endothelial cells derived from hypertensive and normotensive rats. *Am J Physiol.* (1994) 267:H2491–7. 10.1152/ajpheart.1994.267.6.H2491 7528999

[19] Ross R. The pathogenesis of atherosclerosis: a perspective for the 1990s. *Nature.* (1993) 362:801–9. 10.1038/362801a0 8479518

[20] Wenzel P. Monocytes as immune targets in arterial hypertension. *Br J Pharmacol.* (2019) 176:1966–77. 10.1111/bph.14389 29885051 PMC6534790

[21] Mehaffey E Majid DSA. Tumor necrosis factor-α, kidney function, and hypertension. *Am J Physiol Renal Physiol.* (2017) 313:F1005–8. 10.1152/ajprenal.00535.2016 28724611 PMC5668589

[22] Li NF Yao XG Zhu J Yang J Liu KJ Wang YC Higher levels of plasma TNF-alpha and neuropeptide Y in hypertensive patients with obstructive sleep apnea syndrome. *Clin Exp Hypertens.* (2010) 32:54–60. 10.3109/10641960902993087 20144074

[23] Naya M Tsukamoto T Morita K Katoh C Furumoto T Fujii S Plasma interleukin-6 and tumor necrosis factor-alpha can predict coronary endothelial dysfunction in hypertensive patients. *Hypertens Res.* (2007) 30:541–8. 10.1291/hypres.30.541 17664858

[24] Zhang J Patel MB Griffiths R Mao A Song YS Karlovich NS Tumor necrosis factor-α produced in the kidney contributes to angiotensin II-dependent hypertension. *Hypertension.* (2014) 64:1275–81. 10.1161/HYPERTENSIONAHA.114.03863 25185128 PMC4339088

