# Supplementary material for: Circulating MicroRNA-505 May Serve as a Prognostic Biomarker for Hypertension-Associated Endothelial Dysfunction and Inflammation
Source: Front Cardiovasc Med. 2022 Apr 29;9:834121. doi: 10.3389/fcvm.2022.834121 (PMC9099007; doi:10.3389/fcvm.2022.834121)
Supplement: Supplementary file 1 [file Data_Sheet_1.DOCX]

Supplementary Material

**Supplemental Figure 1**

**
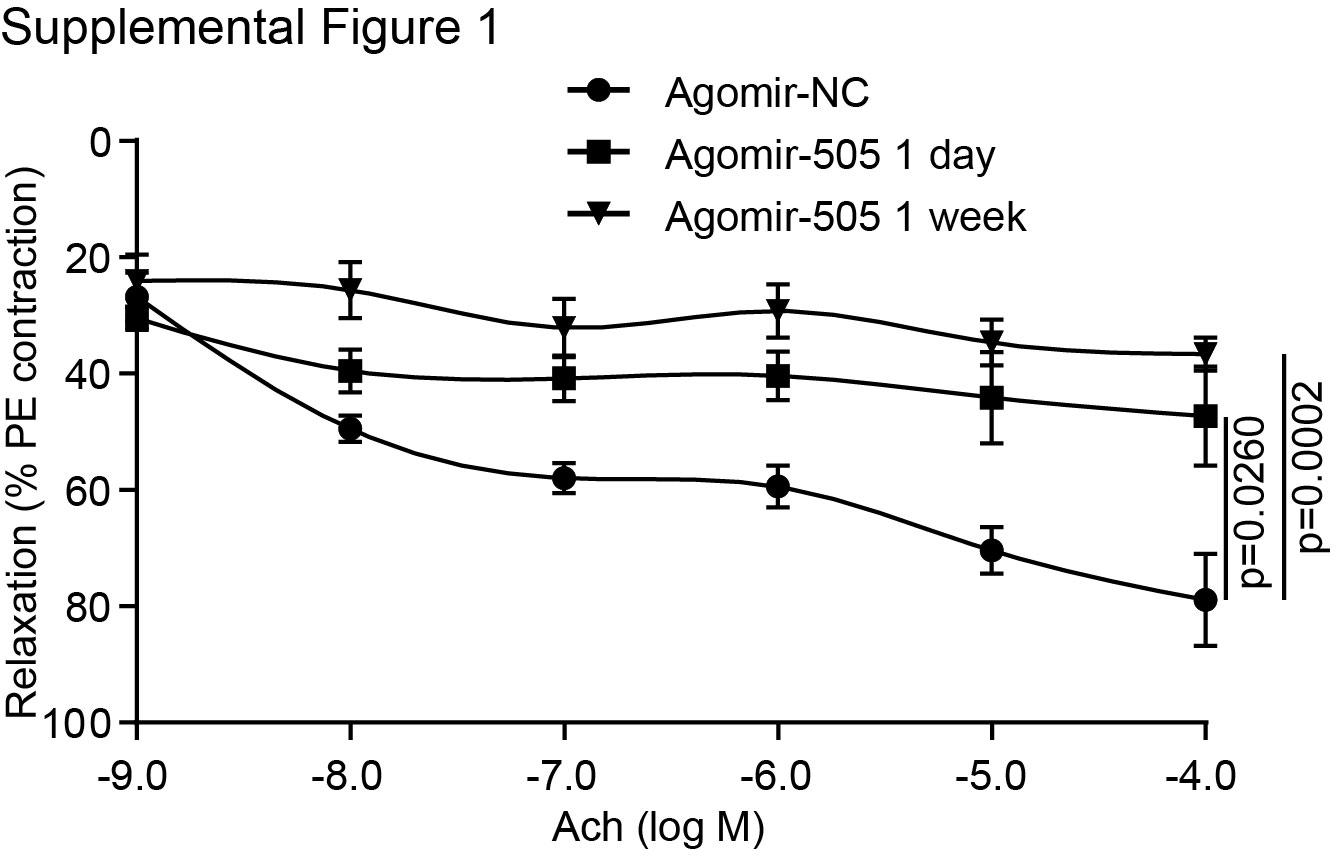
**

**Supplemental Figure 1**. **Elevated circulating miR-505 impairs endothelium-dependent vasorelaxation.** C57/BL6 mice were treated with agomir-NC (10 mg/kg, n=6) or agomir-505 (10 mg/kg, n=6) for 2 weeks through intravenous injection. Aortas were dissected 1 day or 1 week after the termination of agomir-505 or agomir-NC treatment, followed by vascular reactivity assessment. Data represent mean ± SEM. P values are derived from one-way ANOVA and the Student’s t-test.

**Supplemental Figure 2**


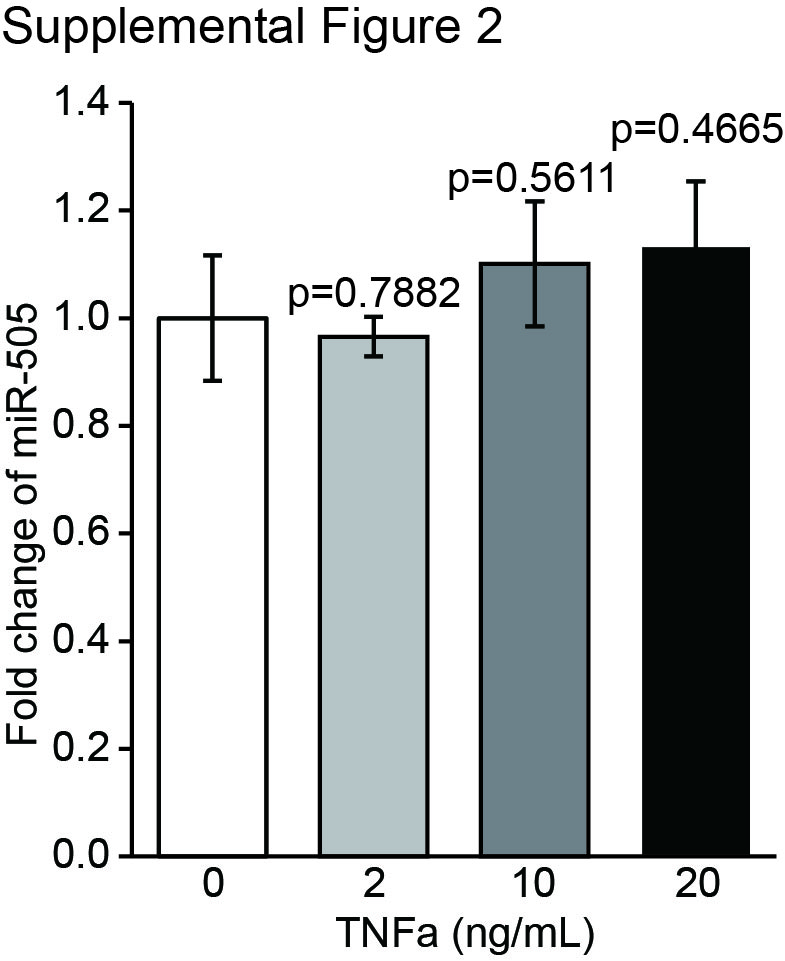


**Supplemental Figure 2. The expression of miR-505 is not altered by TNF-α in HUVECs.** HUVECs were stimulated with TNF-α at 2 ng/ml, 10 g/ml or 20 ng/ml, followed by real-time qPCR analysis of the expression of miR-505. Relative fold change of miR-505 was plotted against that from vehicle-treated cells. Data represent mean ± SEM. P values are derived from one-way ANOVA and the Student’s t-test.

**Supplemental Figure 3**

**
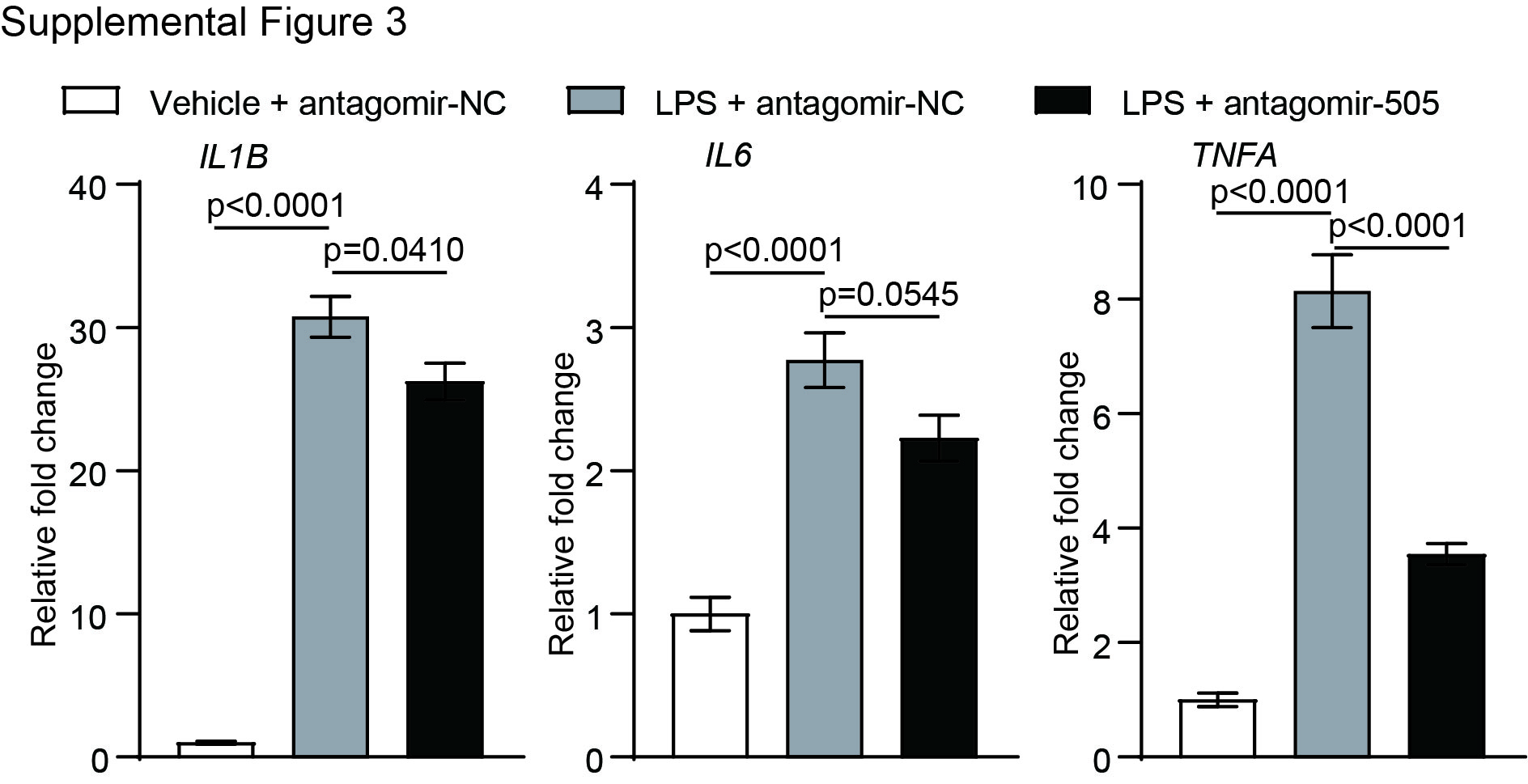
**

**Supplemental Figure 3. Antagomir-505 attenuates LPS-induced expression of proinflammatory genes in HUVECs.** Antagomir-505- or antagomir-NC-transfected HUVECs were exposed to 10 ng/ml LPS stimulation for 4 hr. The expression of *IL1B*, *IL6* and *TNFA* was then analyzed by real-time qPCR analysis. Relative fold change of the gene expression was plotted against that from vehicle-treated antagomir-NC-transfected cells. Data represent mean ± SEM. P values are derived from one-way ANOVA and the Student’s t-test.
